# The role of geospatial hotspots in the spatial spread of tuberculosis in rural Ethiopia: a mathematical model

**DOI:** 10.1098/rsos.180887

**Published:** 2018-09-19

**Authors:** Debebe Shaweno, James M. Trauer, Justin T. Denholm, Emma S. McBryde

**Affiliations:** 1 Department of Medicine, University of Melbourne, Melbourne, Victoria, Australia; 2 Department of Microbiology and Immunology, University of Melbourne, Melbourne, Victoria, Australia; 3 School of Public Health and Preventive Medicine, Monash University, Melbourne, Victoria, Australia; 4 Victorian Tuberculosis Program at the Peter Doherty Institute for Infection and Immunity, Melbourne, Victoria, Australia; 5 Australian Institute of Tropical Health and Medicine, James Cook University, Townsville, Queensland, Australia

**Keywords:** hotspots, transmission, spatial analysis, tuberculosis, metapopulation models

## Abstract

Geospatial tuberculosis (TB) hotspots are hubs of TB transmission both within and across community groups. We aimed to quantify the extent to which these hotspots account for the spatial spread of TB in a high-burden setting. We developed spatially coupled models to quantify the spread of TB from geographical hotspots to distant regions in rural Ethiopia. The population was divided into three ‘patches’ based on their proximity to transmission hotspots, namely hotspots, adjacent regions and remote regions. The models were fitted to 5-year notification data aggregated by the metapopulation structure. Model fitting was achieved with a Metropolis–Hastings algorithm using a Poisson likelihood to compare model-estimated notification rate with observed notification rates. A cross-coupled metapopulation model with assortative mixing by region closely fit to notification data as assessed by the deviance information criterion. We estimated 45 hotspot-to-adjacent regions transmission events and 2 hotspot-to-remote regions transmission events occurred for every 1000 hotspot-to-hotspot transmission events. Although the degree of spatial coupling was weak, the proportion of infections in the adjacent region that resulted from mixing with hotspots was high due to the high prevalence of TB cases in a hotspot region, with approximately 75% of infections attributable to hotspot contact. Our results suggest that the role of hotspots in the geospatial spread of TB in rural Ethiopia is limited, implying that TB transmission is primarily locally driven.

## 1. Introduction

Tuberculosis (TB) is now the world's leading infectious killer with an estimated 10.4 million cases and 1.7 million deaths in 2016. In the same year, TB caused 182 000 cases and 30 000 deaths in Ethiopia [1]. TB demonstrates marked spatial heterogeneity in distribution at any geographical scale and transmission often occurs in households and the general community, leading to the formation of localized transmission hotspots which act as hubs of TB transmission both within and across community groups [2,3]. Consequently, area-based TB control has been recommended rather than conventional contact investigations [4–7] and the identification and targeting of these spatial hotspots has been emphasized to achieve TB elimination goals [8].

In modelling spatial effects on the spread of disease, distinction is made between diffusion (spatially continuous) models and dispersal (metapopulation) models [9]. The first assumes random diffusion of infective individuals into adjacent areas. Dispersal models are used when the considered space is discrete with the population divided into patches [10,11] and assume cross-infection between infective and susceptible populations in different spatial subdivisions [9,12]. Because of spatial diffusion, spatial heterogeneities in disease burden can have important implications for the persistence of infections in a community. Even if disease dies out in some regions, in the presence of spatial structures the coupling between different regions can lead to repeated reintroductions, offsetting local control efforts [9,13].

Several spatial analyses of TB have identified localized transmission hotspots associated with areas of overcrowding and poverty [14–17]. However, the extent to which these geospatial hotspots drive the spatial spread of tuberculosis has not been documented, especially in settings with dispersed population settlements. Although considerable advancements in the methods used to investigate the spatial diffusion of infectious diseases have been made in the last decades [12,18,19], only a few modelling studies were able to apply such methods in the investigation of spatial transmission of TB by incorporating spatial structure. In addition, these studies were limited to overcrowded urban areas [20], and specific cross-border settings [21]. In this study, we aimed to understand the geographical spread of TB from hotspots to regions located at different distances in a remote region of Ethiopia.

## 2. Methods

### 2.1. Identification of spatial hotspots

In our previous spatial analysis of TB in Sheka Zone, a remote region of Ethiopia, we observed considerable spatial heterogeneity [16], with disease clustered in five *kebeles* (the smallest geographical administrative unit in Ethiopia) that contained 20% of the Zonal population but accounted for 53% of notifications. The clusters were identified using local Moran's *I* at 95% confidence interval (electronic supplementary material, figure S1).

### 2.2. Formulation of the cross-coupled metapopulation model

To capture the spatial diffusion of TB from hotspots, we divided the Zonal population into three discrete spatial regions (patches) based on their proximity to hotspots, namely hotspots, adjacent and remote patches. While hotspots constituted areas identified as significant clusters on spatial analysis, adjacent regions comprised regions that share a border with hotspots (electronic supplementary material, figure S1, panel B). *Kebeles* not meeting the criteria for hotspots or adjacent areas were termed remote regions. The average case notification rates per 100 000 population in these patches respectively were 377, 77 and 97 per year.

### 2.3. Model assumptions on spatial coupling

The dynamics of TB were assumed to be identical in the three patches, except for the transmission parameter, which we calibrated on the basis of notification rates in each region. To capture transmission from a hotspot region to the other two regions and vice versa, we developed a cross-coupled metapopulation model by defining contact matrices referred to as WAIFW (‘who acquires infection from whom’) matrices that represent the strength of interaction within and between regions [22] as

$$\left[\begin{array}{ccc} \beta_{11} & \beta_{12} & \beta_{13}\\ \beta_{21} & \beta_{22} & \beta_{23}\\ \beta_{31} & \beta_{32} & \beta_{33} \end{array}\right],$$

where the diagonal elements *β_ii_* (*β*_11_, *β*_22_ and *β*_33_) of the WAIFW matrix represent the average *per capita* effective contact rates per year that an individual in region *i* makes with individuals in region *i*, while the off-diagonal elements (*β_ij_*) represent the average *per capita* effective contact rates per year that an individual in region *j* makes with individuals in region *i*. The degree of coupling between patches is assumed to be smaller than the degree of interaction within patches, such that the values of the off-diagonal elements are found by multiplying the appropriate within-group transmission parameter by the appropriate coupling proportion according to the following four different scenarios considered regarding coupling between regions.

**Figure 1.**
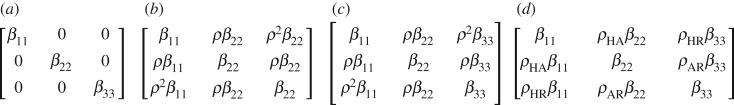
Structure of WAIFW coupling matrices: (*a*) uncoupled model; (*b*) coupled two transmission parameter model; (*c*) coupled three transmission parameter model; (*d*) fully flexible model with *ρ*_HA_: hotspot–adjacent region coupling; *ρ*_AR_: adjacent–remote coupling; *ρ*_HR_: hotspot–remote coupling.

In the first scenario, we considered no coupling between regions (figure 1, model A), with the force of infection determined solely by the prevalence in the index region. Next, we introduced coupling between regions with a single mixing parameter, such that neighbouring regions (i.e. hotspot with adjacent and adjacent with remote) have equal mixing and non-neighbouring regions mix to a lesser degree (i.e. hotspot with remote) (figure 1, models B and C). Third, we introduced coupling between regions with three different coupling parameters, so that each region can exert an independent force of infection on others at different distances (figure 1, model D). These mixing approaches are undertaken either under the assumption of different effective contact rates in each region (figure 1, models C and D) or with only hotspots having a greater transmission parameter (figure 1, model B). In models B, C and D, we assumed that any infective individual in a hotspot region is able to infect susceptible individuals in either of the other two regions and vice versa.

For model D, $\rho_{\mathrm{HA}}$ and $\rho_{\mathrm{AR}}$ are coupling proportions between adjacent regions while $\rho_{\mathrm{HR}}$ is between non-adjacent regions, with $\rho_{\mathrm{HR}}<\rho_{\mathrm{HA}}$. These scaling parameters may take values between 0 and 1, spanning the range from completely uncoupled to maximally coupled systems [12].

The models were then fitted to recorded notification data over 5 years. Best fit solutions were used to find the *β* and *ρ* values. The force of infection on the *i*th group, *λ_i_*, assumes a frequency-dependent transmission and is therefore a weighted sum of infectious TB prevalence in the different spatial groups:

$$\lambda_{i}=\overset{n}{\underset{\;j=1}{\sum }}\frac{\phi \beta_{ij}I_{j}}{N_{j}}.$$

*I_j_* and *N_j_* refer to the number of individuals with active TB and total population in spatial group *j* respectively, while *β_ij_* is the average number of effective contacts per year that an individual in region *j* makes with individuals in region *i*.

**Figure 2.**
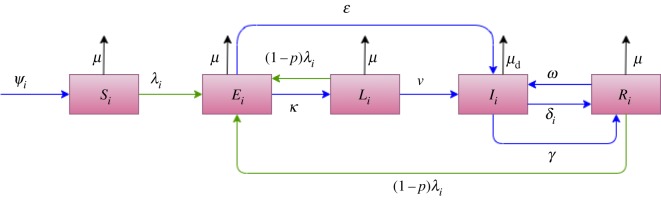
Model structure: blue arrows represent flows between compartments; black represents depletion by mortality; and green represents infection. The subscript *i* takes values from 1 to 3 to index each spatial patch, *p* is the relative risk of infection of a person exposed to TB who has previously been infected (assumed to take values between 0 and 1) and $\lambda_{i}=\sum_{\;j=1}^{n}\left(\phi \beta_{ij}I_{j}/N_{j}\right)$, $\psi_{i}=\mu (S_{i}+E_{i}+L_{i}+R_{i})+\mu_{d}I_{i}$, where *ψ_i_* represents the absolute rate of recruitment to the model for patch *i*, whereas all the other symbols represent the rate of transition out of the compartment from which the arrow originates, which is then multiplied by the value of this compartment.

In this model, the population in each of the three sub-regions is divided into five disjoint states based on TB status, according to a model structure we adapted from a recent publication by Trauer *et al.* [23]. Susceptible (*S*) represents individuals who have never been exposed to *Mycobacterium tuberculosis*, while the early latent (*E*) compartment comprises individuals who have recently been infected (such that progressions to *I* from this compartment correspond to primary TB) [24], and the persistent latent (*L*) compartment represents individuals who were remotely infected but have not yet progressed to active TB. The active TB compartment (*I*) denotes individuals with active TB, while a recovered compartment (*R*) represents individuals who were cured by previous treatment or natural recovery (figure 2).

Susceptible individuals are replenished by births and depleted by infection through contact with infective cases at a rate proportional to the fraction of persons in the active state. Once infected, all individuals transition to the early latent compartment from which they either rapidly progress to active TB (*I*, at rate *ɛ*) or transition to the late latent state (*L*, at rate *κ*). From the late latent state, they may reactivate to join the active TB state (*I*) at a rate *υ*. Once an individual has progressed to active TB (*I*), they either experience natural recovery (*γ*), die from TB-related mortality (*μ*_d_) or are detected and commence treatment (*δ*). Recovered individuals may relapse at rate *ω*, while recovered and latently infected individuals are subject to reinfection at a reduced rate. The rate of non-disease-induced mortality is constant (*μ*), while the additional death rate due to disease affects only class *I* and is also constant (*μ*_d_).

We consider a closed population, with births replacing both TB-related and non-TB-related deaths, such that demographic effects only act to slowly replenish the susceptible pool over time. In the model, only a fraction of diseased individuals comprising all smear-positive and 22% of smear-negative TB patients is considered infectious, in line with a previous report that 17–22% of smear-negative TB cases are infectious [25]. The dynamic transmission model only captures drug-susceptible TB.

The coupling between regions and the rate of flow through compartments is described by the following system of ordinary differential equations:

$$\frac{\text{d}S_{i}}{\text{d}t}=\psi_{i}-\lambda_{i}S_{i}-\mu S_{i},$$

$$\frac{\text{d}E_{i}}{\text{d}t}=\lambda_{i}S_{i}+\;\lambda_{i}(1-p)(L_{i}+R_{i})-(\varepsilon +\kappa +\mu )E_{i}\;,$$

$$\frac{\text{d}L_{i}}{\text{d}t}=\kappa E_{i}-(\lambda_{i}(1-p)+\nu +\mu )L_{i},$$

$$\frac{\text{d}I_{i}}{\text{d}t}=\varepsilon E_{i}+\nu L_{i}+\omega R_{i}-(\delta_{i}+\gamma +\mu_{d})I_{i}$$

$$\frac{\text{d}R_{i}}{\text{d}t}=(\delta_{i}+\gamma )I_{i}-(\lambda_{i}(1-p)+\omega +\mu )R_{i},$$

where

$$\psi_{i}=\mu (S_{i}+E_{i}+L_{i}+R_{i})+\mu_{d}I_{i}$$

$$\lambda_{i}=\overset{n}{\underset{\;j=1}{\sum }}\frac{\phi \beta_{ij}I_{j}}{N_{j}}.$$

The system of ordinary differential equation was solved using a Runge–Kutta algorithm. The numerical solutions were obtained using ode45 in Matlab 2015b.

### 2.4. Model fitting

The metapopulation model was fitted to 5-year TB case-notification data collected from clinical records on all TB patients diagnosed between 2010 and 2014 in health facilities of Sheka Zone, Ethiopia [15]. For model fitting, we aggregated data by patches and year based on patients' places of residence.

When fitting the models to data, we used a Metropolis–Hastings algorithm, with a likelihood function that considered the observed TB notification rate in region *i* and year *y* as the realization of a Poisson process. The expectation of the Poisson distribution (*λ_ij_*) is the mean notification rate from the dynamic TB transmission model at different effective contact rate values within and between regions. The mean notification rate is the flow rate per unit time from the *I* to the *R* compartment through case detection. The site index *i* runs from 1 to 3, representing the 3 patches and the year index *j* runs from 1 to 5 representing 5-year notification data. The Metropolis–Hastings algorithm was initialized by setting initial parameter values and assigning a uniform prior distribution between 0 and 1 for all proportions and all rates for which plausible values were less than 1 (for the parameter set). Then the likelihood of the parameter set given the data was calculated. We then iterated to find the best fitting parameter set, where at each iteration a new candidate parameter set θ* is randomly generated using a multivariate normal proposal distribution centred at the previous accepted parameter set, *θ*. Then the likelihood of the new candidate parameter set θ* given the data *L*(θ*) is determined using the Poisson distribution. The new parameter set θ* is accepted with probability *p* = min(1, *L*(θ*)/*L*(*θ*)). The same procedure was followed for each of the four models and the best fitting model was selected using the deviance information criterion (DIC). DIC assesses models in terms of both their goodness of fit and their parsimony, penalizing models according to the effective number of parameters [26]. We calculated DIC as the sum of the expected posterior value of deviance (−2 times the average of the log-likelihood ratios) and the effective number of parameters in the model, described as the difference between the posterior mean of the deviance and the deviance at the posterior means of the parameters (likelihood ratio evaluated at the average of the parameters) of interest [26]. We ran the model over 120 000 iterations and discarded the first 110 000 as a burn-in. The model was coded in Matlab-R2015b (The MathWorks, 2015) and some of the plots were produced using ggplot2 library in R v. 3.3.1. We collected data used in this study after obtaining ethical approval from the University of Melbourne Health Sciences Human Ethics Subcommittee and the Zonal Health Department of Sheka Zone, Ethiopia.

### 2.5. Simulation of the impact of interventions in hotspots on the spatial spread of tuberculosis

To estimate the extent to which hotspots contributed to TB transmission in the two non-hotspot regions, we first ran the best candidate model to equilibrium using the best fitting parameter values. We subsequently modified the case detection rate only in the hotspots from 65% (baseline value) using increments of 5% to understand the effect this might have on the burden of TB in the two non-hotspot regions.

### 2.6. Parametrization

We considered identical model parameter values across each of the three patches except for case detection rate (table 1). The baseline case detection rate (CDR) of 65% was considered in the hotspot region, while a lower CDR (60%) was considered in the two non-hotspot regions.

**Table 1.** Numerical values of model parameters.

fixed input parameters	value	references	unit
natural mortality rate, *μ*	0.0154	[27]	year^−1^
fast progression rate, *ɛ*	0.4	[28]	year^−1^
stabilization rate, *κ*	3.6	[28]	year^−1^
reactivation rate, *ν*	0.002	[28]	year^−1^
untreated mortality rate, *μ*_d_	0.125	[29]	year^−1^
natural recovery rate, *γ*	0.205	[29]	year^−1^
proportion of incident TB smear-positive	0.33	[30]	proportion
proportion of incident TB smear-negative	0.35	[30]	proportion
case detection rate, *δ*	65%^a^, 60%^b^	[31]	proportion
relapse, *ω*	0.002	[32]	year^−1^
fraction of smear-negative TB infectious	0.22	[25]	proportion
fraction of infectious cases, *ϕ*	0.40	^c^	proportion
protection against infection from latency, *p*	0.79	[33]	multiplier

^a^In hotspots.

^b^In both non-hotspot regions.

^c^The fraction of active cases that are infectious is calculated as the proportion of smear-positive TB (0.33) plus 0.22 times the proportion of smear-negative TB (0.35). The remaining fraction of active cases (0.32) are extrapulmonary and non-infectious.

### 2.7. Goodness-of-fit

We simulated notification data using fitted posterior parameter values to determine whether our model could reproduce the notification data that were previously used to estimate the model parameters. Estimated mean notification rates from the model using fitted parameter values were used as the mean of the Poisson distribution to yield simulated notification rates. We overlayed the observed notification rates on the histograms of simulated notification rates.

## 3. Results

### 3.1. Model comparison

Of the four models, the model assuming equal effective contact rates in adjacent and remote regions (B) was the poorest fitting model based on the DIC, with the simulated data from this model fitting the observed data poorly. The other three models which assumed different contact rates in each patch (A, C, D) demonstrated better fit based on DIC values, including the two models (C and D) which incorporated coupling and the one which did not (A). The model incorporating spatial coupling with a single coupling parameter (C) had a slightly lower DIC than the non-coupled model (A) and the spatially coupled model with separate between-region coupling parameters (D) (table 2). Simulations of notification data based on parameters fitted using models B, C and D produced similar notification rates.

**Table 2.** Credible intervals of estimated parameters and outputs from candidate models. Model A—no coupling; model B—coupled, and similar mixing in the two non-hotspot regions; model C—coupled, with area-specific contact rates; model D—coupled, with area-specific contact rates and three separate coupling terms between regions. *ρ*_HA_: hotspot–adjacent region coupling; *ρ*_AR_: adjacent–remote coupling; *ρ*_HR_: hotspot–remote coupling.

	median (95% CrI) of posterior distributions of model parameters			
parameters	model A	model B	model C	model D
*β*_11_	55.2 (52.8, 58.0)	55.1 (52.6, 57.7)	55.5 (52.9, 58.9)	55.4 (52.8, 57.7)
*β*_22_	14.5 (13.2, 16.1)	14.7 (13.6, 15.8)	2.14 (0.2, 7.4)	8.6 (1.5, 13.6)
*β*_33_	15.3 (13.8, 16.9)	14.7 (13.6, 15.8)	14.7 (13.0, 16.4)	13.6 (11.4, 15.7)
*ρ*_HA_	^a^	0.002 (9 × 10^−5^, 0.006)	0.045 (0.02, 0.06)	0.02 (0.001, 0.05)
*ρ*_AR_	^a^	^a^	^a^	0.06 (0.004, 0.13)
*ρ*_HR_	^a^	^a^	^a^	0.006 (0.0003, 0.016)
notification rate, hotspot	384 (347, 424)	384 (362, 408)	387 (364, 421)	387 (364, 409
notification rate, adjacent	87 (69, 106)	96.7 (84.7, 107.7)	86.2 (74.2, 99.4)	88.2 (75, 102)
notification rate, remote	97 (78, 117)	90.8 (77.6, 103.0)	96.9 (77.7, 114.0)	98.1 (83, 114)
DIC	197	207	195	199

^a^Not applicable.

### 3.2. Parameter estimation

The best model was model C, which assumed different effective contact rates in each region and the proportion of coupling between two non-adjacent regions to be the square of the coupling proportion between adjacent regions. The results of this model are the focus of the rest of this Results section (for outputs from other candidate models, see electronic supplementary material, figures S4–S7). Model C estimated the mean effective contact rates in the hotspot, adjacent and remote regions to be 55.5 (95% credible interval (95% CrI): 52.9, 58.9), 2.2 (95% CrI: 0.2, 7.4) and 14.7 (95% CrI: 13.0, 16.4) per year (table 1). Similarly, this model estimated the strength of coupling between hotspot and adjacent regions to be 4.5% (95% CrI: 2%, 6%), and between hotspots and remote regions to be 0.2%. This means that for every thousand hotspot-to-hotspot transmission events, 45 transmission events occur from hotspot-to-adjacent regions and 2 transmission events occur from hotspot-to-remote regions.

Each of the four models estimated similar effective contact rates in hotspot and remote regions, which is also true for estimated notification rates except for model B. The posterior distributions of estimated parameters from the best fitting cross-coupled metapopulation model (C) were well-fitted by a normal distribution, except for the effective contact rate parameter in the adjacent region that was well-fitted by a gamma distribution (shape parameter = 1.87, scale parameter = 1.19) (electronic supplementary material, figure S2).

**Figure 3.**
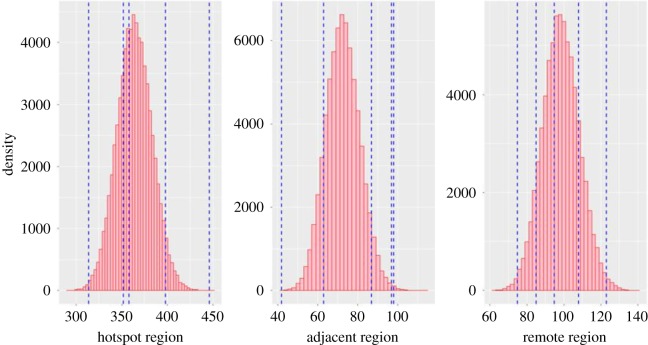
Comparison of simulated notification rate (histograms) and observed notification rates over 5 years (vertical dashed blue lines).

Our model could satisfactorily reproduce the notification rates that were previously used to estimate the set of model parameters (figure 3). We also tested if both the observed and simulated data came from the same underlying distribution using Kolmogrorov–Smirnov test. The test indicated that both the observed data and the simulated data came from the same underlying distribution for all the three patches (hotspots: *D* = 0.36, *p*-value = 0.54; adjacent region: *D* = 0.54, *p*-value = 0.11; remote region: *D* = 0.31, *p*-value = 0.71).

### 3.3. Analysis of covariance of fitted model parameter posterior probability distributions

**Figure 4.**
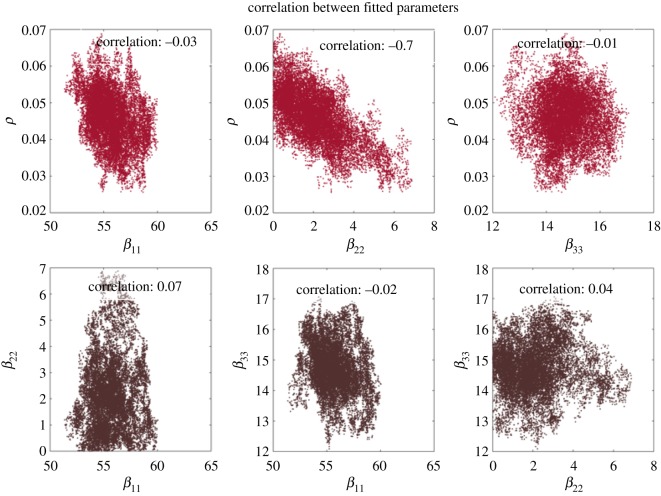
Correlation between the coupling terms and effective contact rates by region.

We further assessed the behaviour of model C by examining the relationship between parameter pairs. The effective contact rates in hotspot (*β*_11_) and remote regions (*β*_33_) were not correlated with the coupling term (*ρ*). However, the number of effective contacts in an adjacent region (*β*_22_) and the coupling term (*ρ*) demonstrated a strong negative correlation (correlation coefficient: −0.7) (figure 4). The presence of considerable correlation between *β*_2_ and *ρ* reduced the precision of the estimates for these two parameters.

We further assessed the behaviour of model C by examining.

### 3.4. Simulation of the impact of improving case detection in hotspots on the neighbouring regions

**Figure 5.**
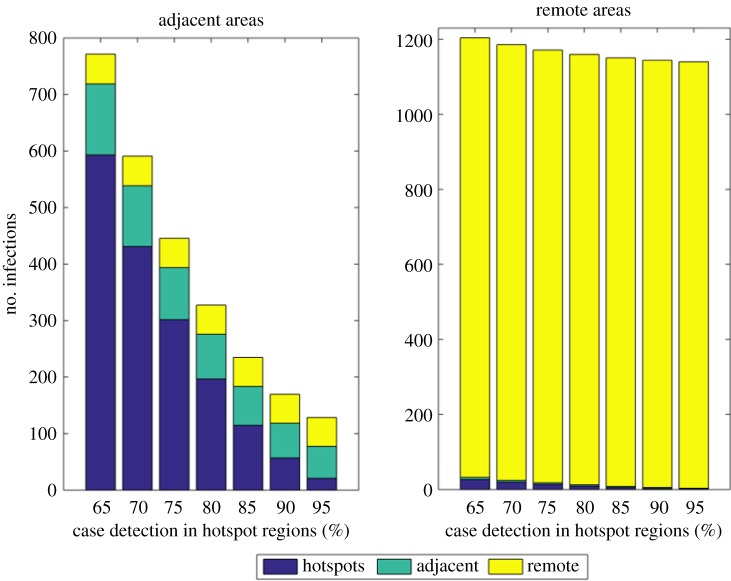
The number of infections in the two non-hotspot regions attributable to mixing with hotspots.

In a cross-coupled metapopulation formulation, infection in a given patch is a function of disease prevalence in all sub-regions (patches) and the degree of coupling between and within regions. Thus, although the extent of spatial coupling between hotspots and the other two regions is weak, the proportion of TB infection in an adjacent region due to mixing with hotspots was about 76% (95% CrI: 52%, 92%,); and was 2.5% (95% CrI: 0.7%, 3.2%) in the remote regions in the first 5 years at a baseline CDR in hotspots (figure 5). That is, out of 771 (95% CrI: 258, 1736) secondary infections in the adjacent region, 593 (95% CrI: 237, 904) were generated due to mixing with hotspots. As a result, improving CDR in the hotspot regions has significant impact on the neighbouring region. For instance, the proportion of infections in the adjacent regions due to mixing with hotspot regions drops by 10% down to 65% (95% CrI: 37%, 86%) when case detection rate in the hotspot regions goes from 65% (the baseline) to 70%. Similarly, when case detection rate goes from 65% to 95%, the proportion of infections in the adjacent regions due to mixing with hotspot regions drops down to 16% (95% CrI: 7%, 39%). However, the effect of the same intervention is only marginal in the remote regions (figure 5). This is because of extremely large number of prevalent infectious cases in hotspots who are available to make cross-contact.

## 4. Discussion

Using cross-coupled metapopulation models, we quantified the role of TB hotspots in the spatial spread of TB in rural Ethiopia, demonstrating that spatial coupling between TB hotspots and the surrounding regions is limited. However, despite limited mixing between hotspots and adjacent regions, the very high rate of transmission in hotspots means that they contribute significantly to disease in immediately adjacent surrounding areas.

Of the models considered, the model that assumed spatial coupling with different effective contact rates by region attained the best combination of accuracy and parsimony, although a similar model with different transmission rates by region but without spatial coupling also demonstrated a reasonable fit to data. The best fitting model predicted 45 hotspot-to-adjacent region transmission events and two hotspot-to-remote region transmission events for every 1000 hotspot-to-hotspot transmission events, although it was not possible for us to confirm this with additional data on mobility or organism genotypes. However, we consistently predicted a coupling proportion of less than 10% between adjacent regions using all of our cross-coupled models.

In our models, the extent of population mixing between regions was modest, implying that the probability of contact with externals is much lower than the probability of local contact and that TB transmission in rural Ethiopia is predominantly locally driven. This is consistent with our expectations, as the study was conducted in a rural area with a dispersed population and limited long-distance population movement (most population movements are by foot), as well as its close fit to data. Thus, TB control efforts targeting hotspots in rural Ethiopia may not achieve the anticipated impact on community-wide TB control, although this is the subject of further investigation.

Although coupled metapopulation models are standard approaches in the presence of heterogeneity, there is no universally accepted approach to quantifying epidemiological coupling between different regions [9,18,19]. Previous works (mainly of measles) have used different approaches: considering a range of coupling parameters [12], estimating coupling by trial and error from simulation [34] or using intuition alone. For measles, the coupling term has been estimated between 10^−4^ and 10^−1^ in higher resource settings [12,18,19]. In common with many of the studies described above, our study used a simulation approach by developing an algorithm to find the best fitting coupling term and transmission parameters. However, the strong correlation between the transmission term in adjacent regions and the coupling term might have reduced the amount of information available on cross-coupling. Thus, in the presence of trade-off between the effective contact rate in adjacent regions and the coupling term, the accurate interpretation of the extent of coupling or the effective contact rate in the adjacent region remains a challenge. Future works would require genotypic data to validate the extent of transmission from hotspots to neighbouring communities, although this information is often unavailable in high incidence settings.

Our coupling term is considerably lower than hotspot-to-community transmission term used in a modelling study of urban Rio de Janeiro, in which each infective in a hotspot region was assumed to cause 0.5 transmission events outside of the hotspot for each event caused within the hotspot [20]. However, it is important to note that this parameter in the Rio de Janeiro study was derived from a study that was based on a very small sample size (*n* = 10) and geographical clustering was not statistically defined [35]. Rather, geographical clusters were defined by at least two cases sharing a common molecular structure and from the same or close neighbourhoods. Moreover, it should be noted that these urban settings could be much more strongly coupled than the broad geographical region considered in this study, such that these estimates may both be accurate.

Recent works have recommended quantification of coupling terms by measuring population mobility patterns [19]. An important modelling study describing the impact of cross-country border population mobility on TB burden in a low incidence setting concluded that TB in the Australian Torres Strait region is driven by the TB dynamics in Papua New Guinea [21]. This was not unexpected given that 98% of the mobility is to Australia for cultural practices as well as for healthcare. Similarly, another cross-border study also suggested that expanding the directly observed treatment short course programme in the neighbouring high incidence settings (Mexico, Haiti and Dominican Republic) could reduce TB-related morbidity and mortality among migrants to the United States [36]. In contrast, in our study setting, there is no similar reason to assume large population movements between rural regions which do have similar features in terms of healthcare access.

Although the extent of coupling observed in this study is low (less than 5%), the proportion of infections in the adjacent region due to mixing with hotspots is considerable (more than 75%). This emerges from the proportional relationship between the coupling term and intra-region mixing (prevalence), with the large number of infectious cases in a hotspot region relative to the adjacent regions leading to a considerable contribution to infection in the adjacent region. This is consistent with a previous analysis indicating that infectious disease persistence in a community can be due to either high intragroup mixing, strong coupling or both [13]. Thus, TB control efforts in extremely high transmission regions may provide additional TB risk reduction to surrounding regions.

Our study has important limitations. Our classification of the study region into three spatially discrete groups may be overly simplistic, although increasing the number of patches simulated would present additional challenges in fitting many parameters. More complex models, such as spatially continuous models or agent-based models, may be useful to investigate these links at finer spatial scales. In addition, the model presented here assumes only a single strain of drug-sucseptible TB in this setting where drug susceptibility testing is unavailable. Considering multidrug-resistant TB, HIV and urban dynamics into the model is limited by available data, but will be the subject of future research.

## 5. Conclusion

Our study suggests that TB in rural Ethiopia is primarily driven by local transmission, rather than spillover from hotspot regions. However, the epidemiology of tuberculosis in regions adjacent to transmission hotspots is considerably contributed by these hotspots. Control efforts in high transmission regions may provide some additional TB risk reduction in surrounding regions, although locally focused measures remain essential.

## Supplementary Material

Supplementary Material

## Data accessibility

Data and the Matlab code used are available from the Dryad Digital Repository: http://dx.doi.org/10.5061/dryad.fg3js19 [37].
