# Supplementary material for: The role of geospatial hotspots in the spatial spread of tuberculosis in rural Ethiopia: a mathematical model
Source: R Soc Open Sci. 2018 Sep 19;5(9):180887. doi: 10.1098/rsos.180887 (PMC6170575; doi:10.1098/rsos.180887)
Supplement: Supplementary Material [file rsos180887supp1.docx]

**Supplementary Material**

The role of geospatial hotspots in the spatial spread of tuberculosis in rural Ethiopia: A mathematical model

Debebe Shaweno, James M Trauer, Justin T Denholm, Emma S McBryde

**Spatial TB clusters in Sheka Zone, Ethiopia**


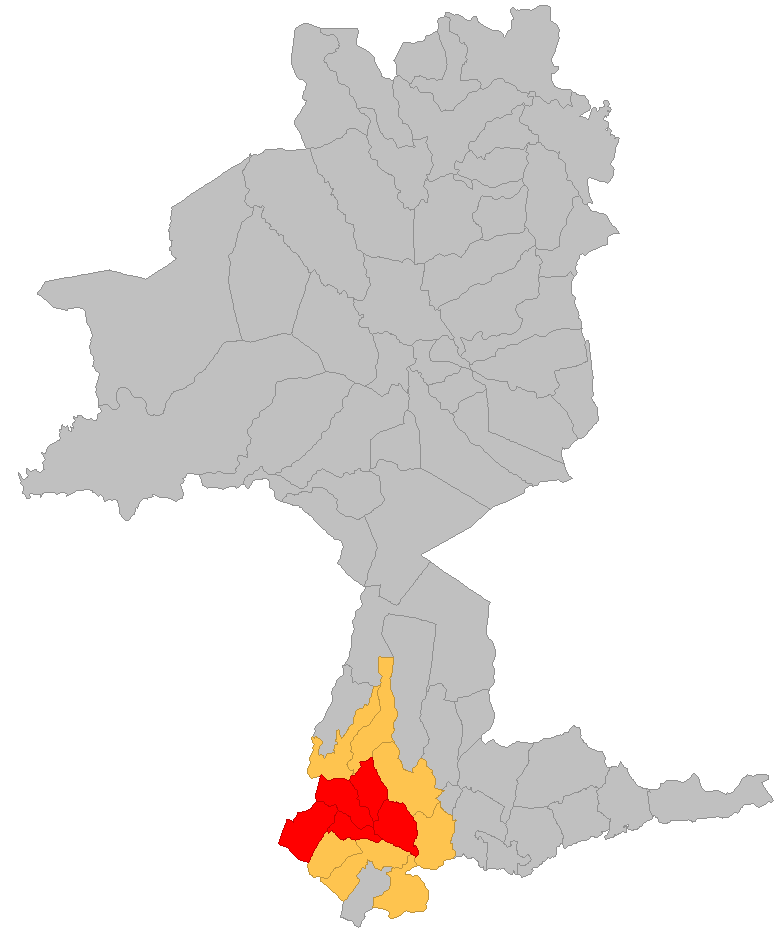


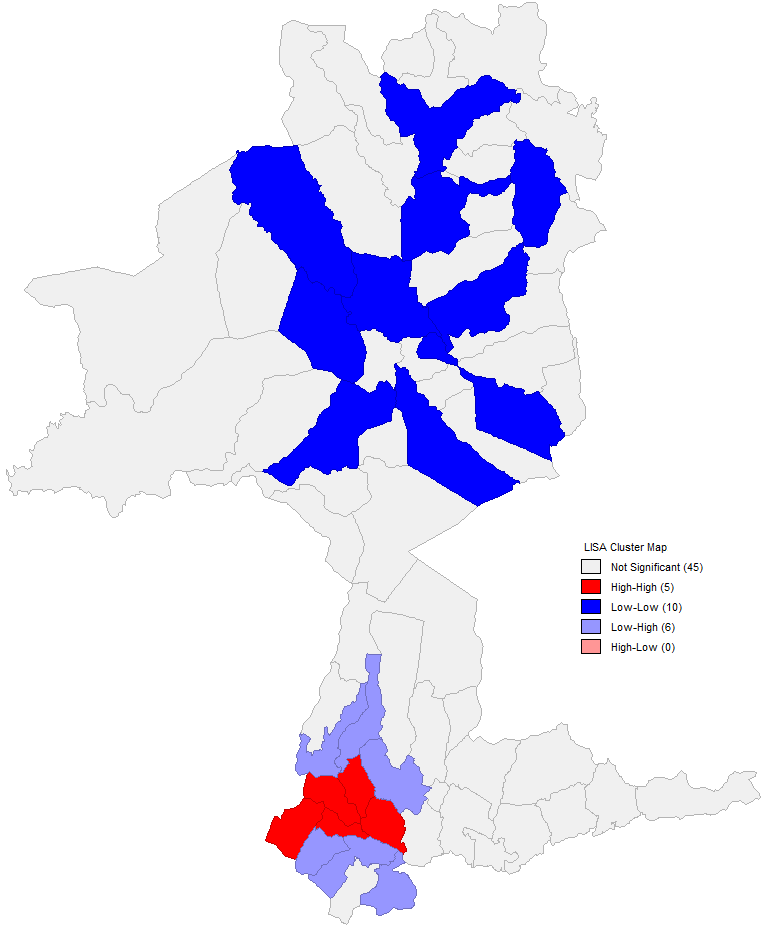


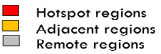


Figure 1: Spatial distribution of TB in Sheka Zone, 2010-2014. Panel A shows the spatial clustering according to local Moran’s I, where High-High (also called hotspot) indicates that high incidence regions are adjacent to high incidence regions. Similarly, Low-Low indicates that areas with significantly lower diseases burden are neighbouring regions with significantly low values. High-Low or low high clusters indicate spatial outliers where high incidence regions are adjacent to low incidence regions. Panel B shows the spatial metapopulation structure used in this analysis.

**Deviance information criterion**

In this study, we computed the deviance information criterion as follows based on an approach presented in Spiegelhalter et al. (2002):

Deviance was defined as -2 times the loglikelihood and can be written as:

D (*y, θ*) = -2log *p (y| θ)* …………………………………………..[1]

Having defined deviance as above, the deviance information criterion (DIC) can be computed as follows:

DIC= $\bar{D}$ + PD ……………………………………………………… [2]

$PD=E_{\theta|y}\left[ D \right]-D(E_{\theta|y}[\theta])$ …………………………………… [3]

= $\bar{D}-D\left( \bar{\theta} \right)$………………………………………………….... [4]

Where,$\bar{D}$ is the expected posterior value of the deviance (the average of log-likelihood ratios) and PD is the effective number of parameters in the model. PD is calculated as the difference between the posterior mean of the deviance and the deviance at the posterior means of the parameters (likelihood ratio evaluated at the average of the parameters) of interest. Adding the PD to the posterior mean deviance ($\bar{D}$) gives a *deviance information criterion* for comparing models [2].

Models with the lowest DIC estimates indicate models with a better performance, although there is no agreement on the magnitude of the difference between different DIC values that constitutes a meaningful difference. A general rule of thumb suggests that differences of more than 10 should definitely rule out the model with the higher DIC, while differences between 5 and 10 are substantial. However, if the difference is less than 5 and if different inferences would be drawn from the models being compared, then it is misleading to report the model with the lowest DIC only [3].

**Calculation of the likelihood**

In this analysis we used Poisson likelihood in the model fitting as follows:

$$Likelihood=\prod_{i=1}^{3} \prod_{j=1}^{5} \frac{\lambda^{k}e^{-\lambda}}{k!}$$

The subscript *i* takes values from 1 to 3 to index each spatial patch, and the subscript j takes values from 1 to 5 to index years. κ refers to the observed notification and λ represents the expected Poisson mean, which is the model estimated notification.

**Output from the best fitting model**


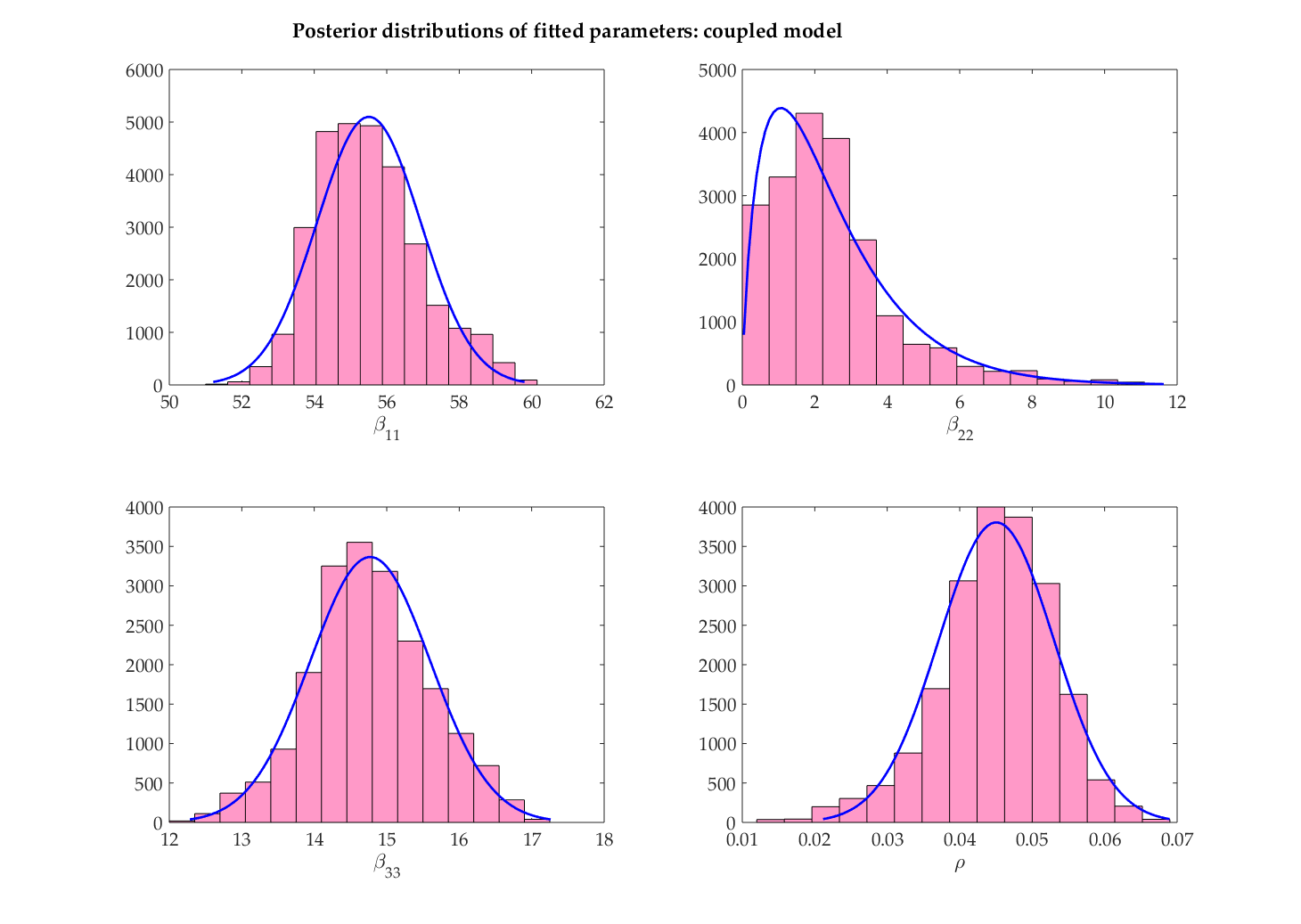


μ= 55.3

σ= 1.5

μ= 0.046

σ= 0.007

μ= 14.7

σ= 0.85

α= 1.87

β= 1.19

**Figure 2:** Posterior distribution of fitted parameters under the assumption of mixing with different contact rates in each region (μ- mean, σ- standard deviation parameters of normal distributions; α and β are the shape and scale parameters of a gamma distribution).


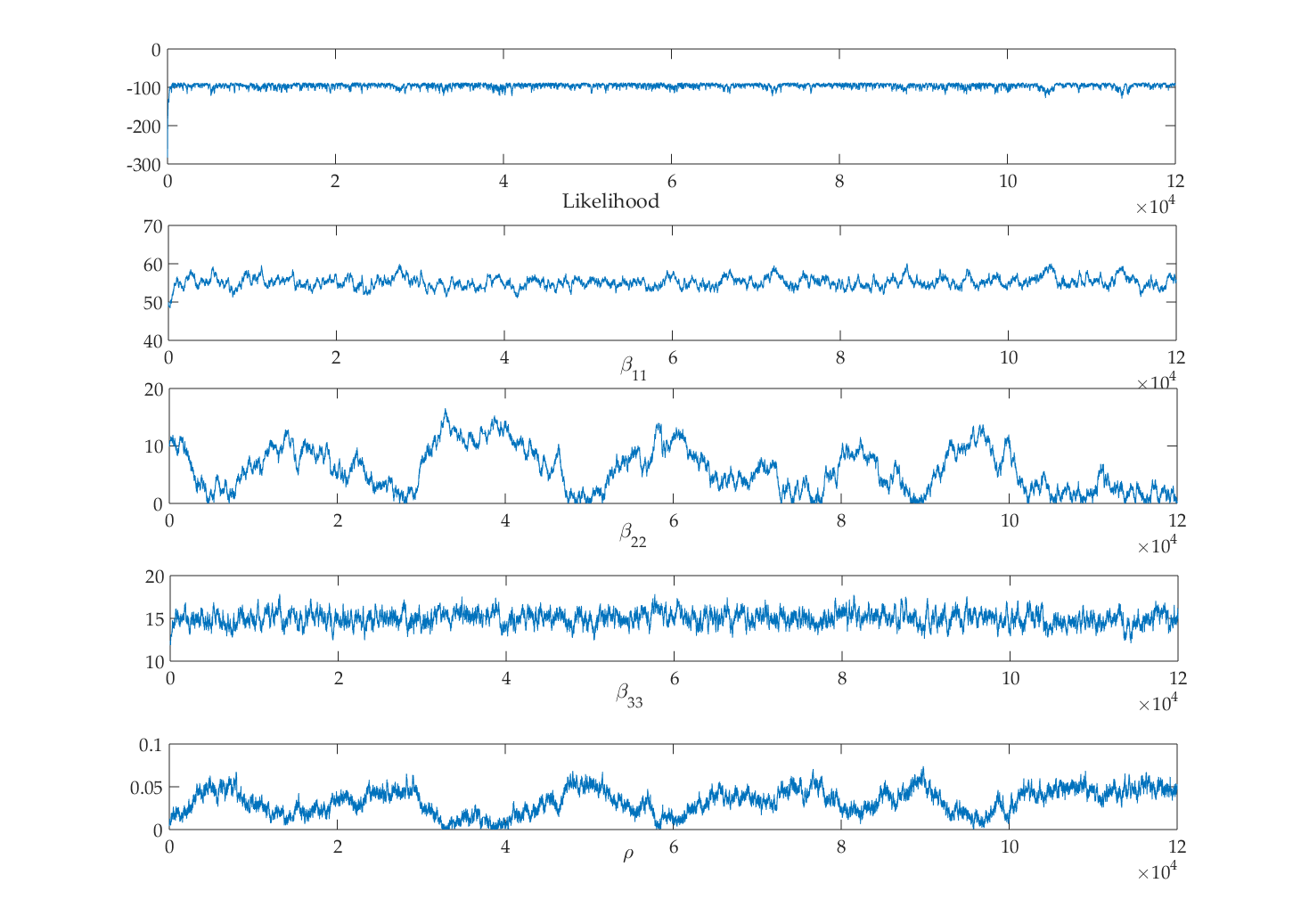


**Figure 3: Sample trace plots of estimated parameters and the likelihood from the best fitting model.**

**Outputs from the candidate models**

1. **Model A: non- spatially coupled model (ρ**$\boldsymbol{=0}$**)**

**
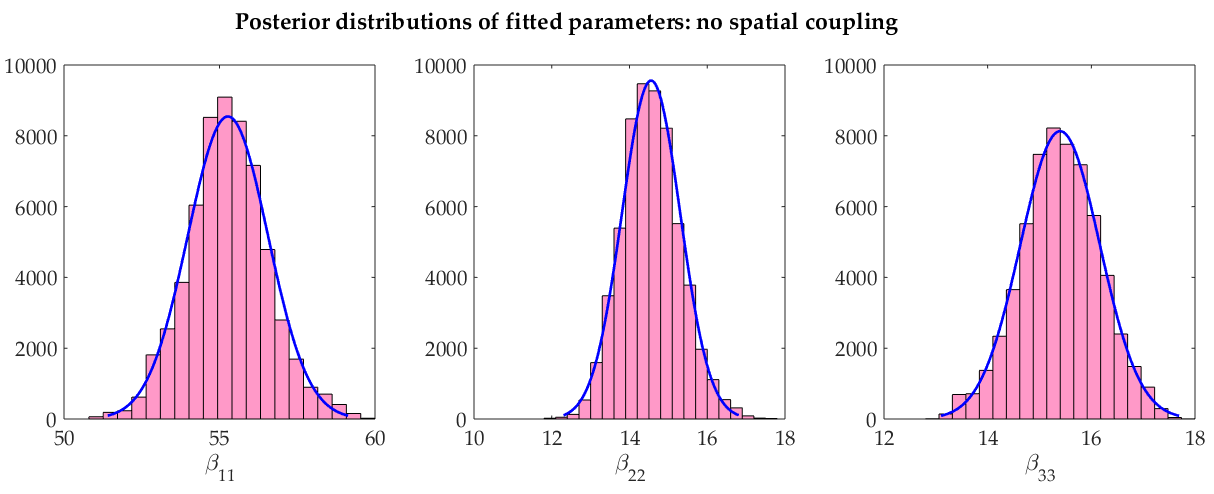
**

Figure 4. Posterior distribution of fitted parameters; β_11_-hotspot, β_22_-adjacent, β_33_- remote region

1. **Model B: Coupled model with equal contact rates in the two non-hotspot regions**


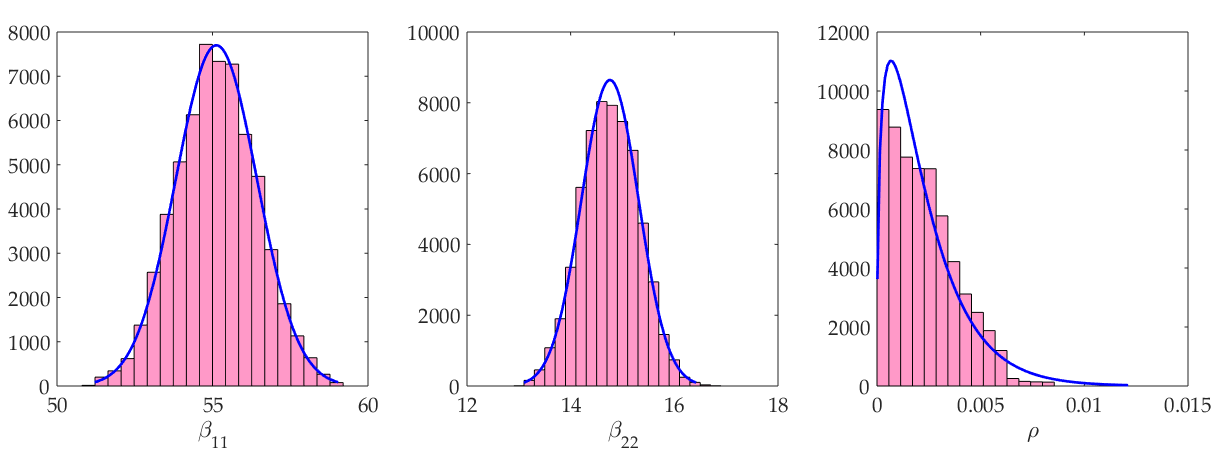


Figure 5. Posterior distribution of fitted parameters; β_11_-hotspot, β_22_-adjacent & remote, ρ- coupling term

1. **Model D: Spatially coupled model with separate coupling parameters across different regions**

**
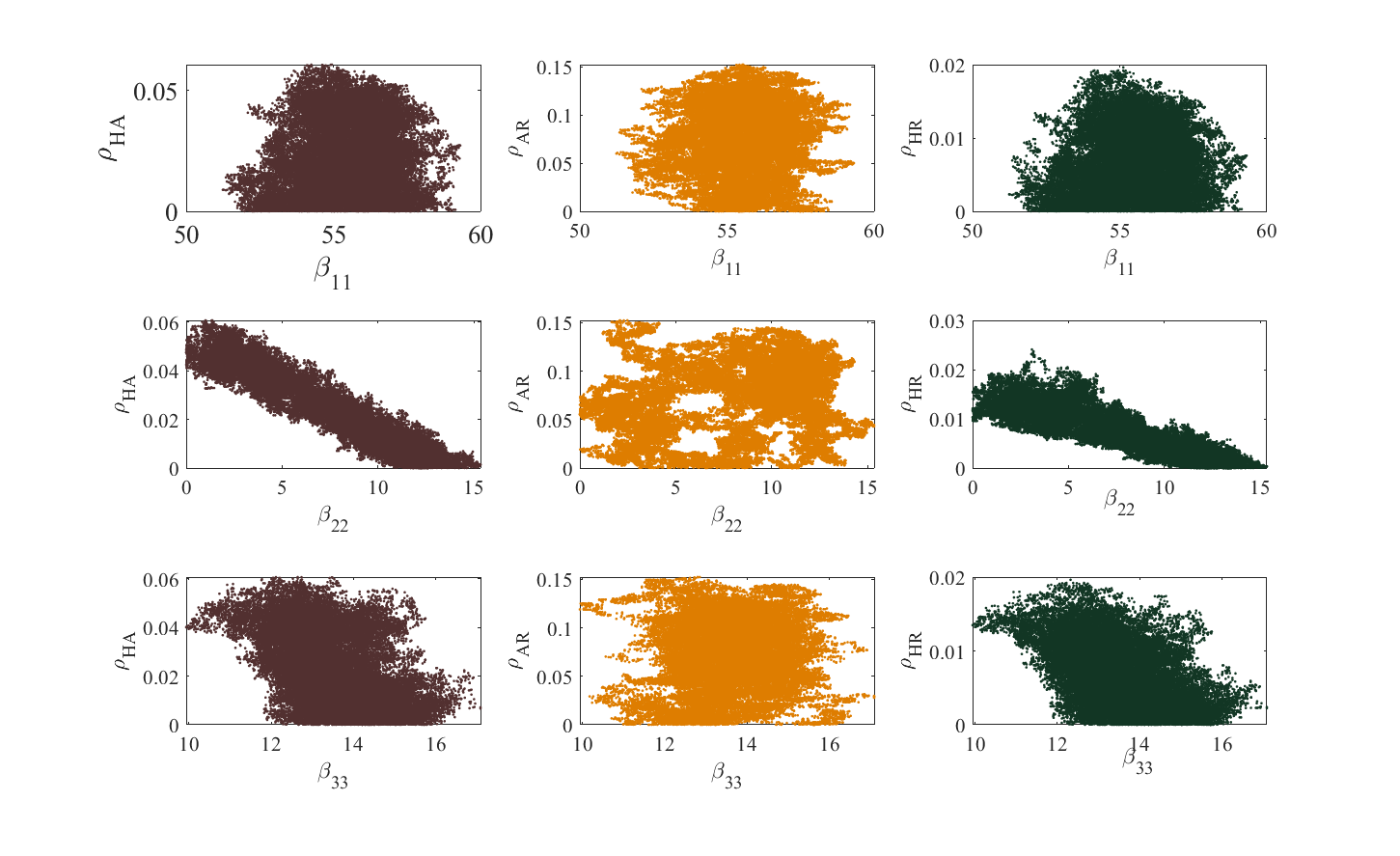
**

**Figure 6: Correlation between pairs of parameters of model D**

**Figure 7. Posterior distribution of parameters, model D**

1. Gelman A, Carlin JB, Stern HS, Rubin DB: **Bayesian data analysis, 2nd edn. Texts in Statistical Science**. In*.*: Boca Raton, London, NewYork, Washington DC: Chapman & Hall, CRC; 2004.

2. Spiegelhalter DJ, Best NG, Carlin BP, Van Der Linde A: **Bayesian measures of model complexity and fit**. *Journal of the Royal Statistical Society: Series B (Statistical Methodology)* 2002, **64**(4):583-639.

3. **DIC: Deviance Information Criteria** [<https://www.mrc-bsu.cam.ac.uk/software/bugs/the-bugs-project-dic/>]
